# In Vitro Evaluation of Bacterial Adhesion and Bacterial Viability of *Streptococcus mutans*, *Streptococcus sanguinis*, and *Porphyromonas gingivalis* on the Abutment Surface of Titanium and Zirconium Dental Implants

**DOI:** 10.1155/2019/4292976

**Published:** 2019-06-13

**Authors:** Ana Stefany Meza-Siccha, Miguel Angel Aguilar-Luis, Wilmer Silva-Caso, Fernando Mazulis, Carolina Barragan-Salazar, Juana del Valle-Mendoza

**Affiliations:** ^1^School of Dentistry, Faculty of Health Sciences, Universidad Peruana de Ciencias Aplicadas, Lima, Peru; ^2^School of Medicine, Research and Innovation Center of the Health Sciences Faculty, Universidad Peruana de Ciencias Aplicadas, Lima, Peru; ^3^Laboratorio de Biología Molecular, Instituto de Investigación Nutricional, Lima, Peru

## Abstract

**Objective:**

To evaluate the in vitro adherence and viability of 3 bacterial species *Streptococcus mutans* (ATCC 25175), *Streptococcus sanguinis* (ATCC 10556), and *Porphyromonas gingivalis* (ATCC 33277) on the surfaces of dental implants of titanium, zirconium, and their respective fixing screws.

**Methods:**

Two analysis groups were formed: group 1 with 3 titanium pillars and group 2 with 3 zirconium pillars, each with their respective fixing screws. Each of these groups was included in tubes with bacterial cultures of *Streptococcus mutans* (ATCC 25175), *Streptococcus sanguinis* (ATCC 10556), and *Porphyromonas gingivalis* (ATCC 33277). These samples were incubated at 37°C under anaerobic conditions. Bacterial adherence was assessed by measurement of the change in colony-forming units (CFU), and bacterial viability was evaluated with the colorimetric test of 3-(4,5-dimethylthiazol-2)-2,5 diphenyl tetrazolium bromide (MTT).

**Results:**

The bacterial adhesion in the titanium abutments was higher for *Streptococcus mutans* (190.90 CFU/mL), and the viability was greater in *Porphyromonas gingivalis* (73.22%). The zirconium abutment group showed the highest adherence with *Streptococcus mutans* (331.82 CFU/mL) and the highest bacterial viability with the *S. sanguinis* strain (38.42%). The titanium fixation screws showed the highest adhesion with *S. sanguinis* (132.5 CFU/mL) compared to the zirconium fixation screws where *S. mutans* had the highest adhesion (145.5 CFU/mL). The bacterial viability of *S. mutans* was greater both in the titanium fixation screws and in the zirconium fixation screws 78.04% and 57.38%, respectively.

**Conclusions:**

Our results indicate that there is in vitro bacterial adherence and viability in both titanium abutments and zirconium abutments and fixation screws for both. *Streptococcus mutans* is the microorganism that shows the greatest adherence to the surfaces of both titanium and zirconium and the fixing screws of the latter. On the contrary, bacterial viability is greater on the titanium abutments with *P. gingivalis* than on the zirconium abutments with *S. sanguinis*. With respect to the fixation screws, in both cases, the viability of *S. mutans* was greater with respect to the other bacteria. In general, the titanium abutments showed less adherence but greater bacterial viability.

## 1. Introduction

A biofilm is considered a bacterial functional community made up of one or more species of microorganisms attached to a solid surface. The pathogenesis of periodontal inflammation begins with the colonization of pathogenic bacteria in a susceptible host, although other environmental factors also play a role in the development of disease [1]. The accumulation of bacterial plaque is required for the development of periodontal inflammation and is also an essential step in other periodontal pathologies [2]. According to Socransky [3], specific bacteria have niche locations of colonization within the oral cavity, and their characteristics are subdivided into primary and secondary colonizers. We have included *Streptococcus mutans* and *Streptococcus sanguinis* in our study because the *Streptococcus* spp. are considered primary colonizers. We have also included *Porphyromonas gingivalis*, a secondary colonizer [3], because of its strong association with peri-implantation pathologies [4].

The conventional dental implant is a two-piece implant that consists of a root component known as the implant and the abutment. This procedure is considered the most successful management for the replacement of missing teeth [5]. When a disequilibrium between pathogenic and nonpathogenic bacteria in the oral microbiota occurs, there is a subsequent increase in adherence of bacteria and therefore an increase in the risk of periodontal infection, most commonly peri-implant mucositis and peri-implantitis [6].

Multiple factors are involved in the pathogenesis of peri-implant disease, including systemic disease like diabetes [7], a previous history of tobacco [8], or periodontitis [9]. However, despite the multifactorial etiology of peri-implant infections, a common denominator lies in the fact that the dental implant must be colonized with specific bacteria before disease onset. Finding the bacterial adhesion and viability of different material abutments will aid in the etiologic understanding of the disease [10].

A previous meta-analysis showed that the prevalence of peri-implantitis was 9.83% and the prevalence of peri-implant mucositis was 29.48%. [11] Bacterial adherence on conventional dental implants is the primary cause for the development of peri-implanting mucositis and peri-implantitis. The characteristics of the surface of the dental abutment will contribute to the adherence of microorganisms [12, 13].

The objective of this study is to evaluate the adherence and viability of *Streptococcus mutans*, *Streptococcus sanguinis*, and *Porphyromonas gingivalis* when exposed in vitro to the surface of zirconium and titanium abutments and fixing screws.

## 2. Materials and Methods

### 2.1. Samples

Our sample included 6 bacterial cultures with different strains of *Streptococcus mutans* (ATCC 25175), *Streptococcus sanguinis* (ATCC 10556), and *Porphyromonas gingivalis* (ATCC 33277) with abutments of two different materials, titanium, and zirconium.

The dental abutments of titanium and zirconium were acquired from Biohorizons®, and the bacterial strain samples of *Streptococcus mutans* (ATCC 25175), *Streptococcus sanguinis* (ATCC 10556), and *Porphyromonas gingivalis* (ATCC 33277) were obtained from Gen Lab in Peru, a representative of MicroBiologics® (USA). The exclusion criteria in this study included abutments of titanium or zirconium with rugose surfaces, with irregular cuts or those not sealed correctly.

We proceeded to sterilize the materials for 15 minutes under UV light inside a laminar flow cabin type II.

### 2.2. Bacterial Culture

The bacterial samples of *Streptococcus mutans*, *Streptococcus sanguinis*, and *Porphyromonas gingivalis* were cultured independently in blood agar plates with supplemental 10% sterile bovine blood. The culture was carried out following the manufacturer's instructions. The plates containing the bacteria were incubated in an Anaerocult® and Anaerotest® controlled anaerobic chamber at 37°C for 10 days in the case of *Porphyromonas gingivalis* and for 3 days in the case of *Streptococcus mutans* and *Streptococcus sanguinis*.

### 2.3. Cultures to Evaluate Adhesion and Bacterial Viability

The titanium and zirconium abutments and their corresponding fixing screws were placed on a sterile Petri dish with 24 pits (Falcon Plastics, Oxnard, CA), and 1000 *µ*L of a bacterial suspension with a 0.5 McFarland scale density was added to each pit. The samples were then incubated at 37°C for 72 hours under controlled anaerobic conditions. The bacterial adhesion and bacterial viability were evaluated once the incubation period was over.

The bacterial adhesion was evaluated by measuring colony-forming units (CFU). Serial dilutions were made in order to obtain the lower quantity of bacteria in the sample. Subsequently, a plate dissemination method was utilized, and a direct microscopic count of CFU was done for each sample [14, 15].

The bacterial viability was evaluated by determining the absorbance values measured by an ELISA reader (Bio-Rad) following colorimetric MTT tests based on the reduction of mitochondrial enzymes [16].

## 3. Results

### 3.1. Adherence and Bacterial Viability on the Surface of Titanium Abutments and Fixing Screws

The bacterial adherence was determined using CFU measured by direct microscopic count. The in vitro evaluation of titanium abutment showed the highest bacterial adherence (190.90 CFU/mL) with *Streptococcus mutans* followed by adherence values of 167.5 and 153.9 CFU/mL for *S. sanguinis* and *P. gingivalis*, respectively. In relation to the bacterial viability, *P. gingivalis* showed the highest value with 73.22% while *S. mutans* and *S. sanguinis* showed bacterial viability of 55.37% and 52.58%, respectively.

**Figure 1:**
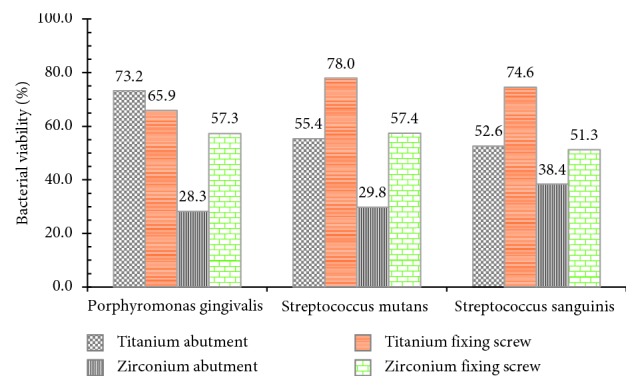
Bacterial viability of *Streptococcus mutans* (ATCC 25175), *Streptococcus sanguinis* (ATCC 10556), and *Porphyromonas gingivalis* (ATCC 33277) on the abutment surface and fixing screw of titanium and zirconium.

The fixing screw had the highest bacterial adherence for *S. sanguinis* (132.5 CFU/mL) and the highest bacterial viability with *S. mutans* (78.04%) (Figure 1).

### 3.2. Adherence and Bacterial Viability on the Surface of Zirconium Abutments and Fixation Screws

The in vitro evaluation of the zirconium abutment showed the highest bacterial adherence for *S. mutans* with 331.82 CFU/mL followed by *S. sanguinis* and *P. gingivalis* with values of 135 CFU/mL and 228.80 CFU/mL, respectively. In relation to the bacterial viability, *S. sanguinis* had 38.42% followed by *S. mutans* with 29.82% and *P. gingivalis* with 28.26%.

**Figure 2:**
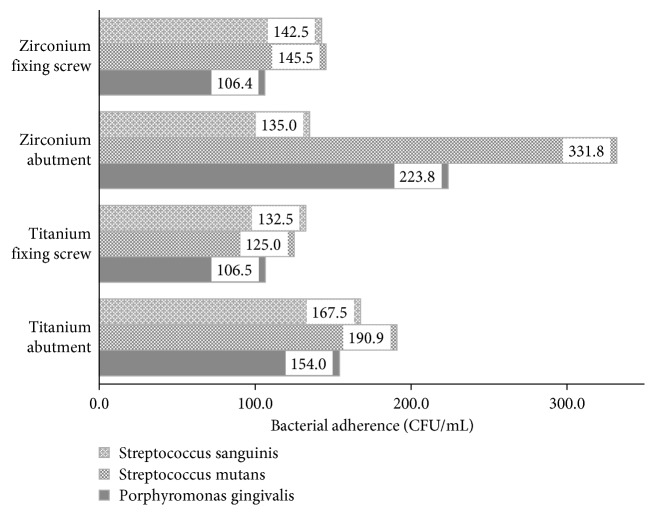
In vitro evaluation of bacterial adherence of *Streptococcus mutans* (ATCC 25175), *Streptococcus sanguinis* (ATCC 10556), and *Porphyromonas gingivalis* (ATCC 33277) on the surface of zirconium/titanium abutment dental implants and fixing screw (CFU = colony-forming units).

The results of the fixation screw showed a similar adherence to *S. mutans* (145.45 CFU/mL), *S. sanguinis* (142.5 CFU/mL), and *P. gingivalis* (106.5 CFU/mL). The values for bacterial viability also showed some similarities with *S. mutans* and *S. sanguinis* with values of 57.38% and 57.33%, respectively. *P. gingivalis* had the lowest bacterial viability with 51.31% (Figure 2).

## 4. Discussion

The oral cavity has a particular bacterial population with the capacity to form biofilms, which allows it to coexist with the tissues that surround them. Oral bacteria are established and grow in situ because they achieve adhesion to the hard surface of the teeth and soft epithelial tissues [17–19]. These characteristics are of special importance in the current implantology because the implant constitutes a key element for an adequate and sustainable osseointegration in the process of restoring a tooth that was extracted due to caries, disruptive periodontal disease, or agenesis [20–23]. In this context, inflammatory diseases, including infections that affect the soft and hard tissues surrounding an implant, represent a challenge in the search for strategies for decontamination of implant surfaces and identification of the bacteria that can colonize these structures in order to maintain a healthy interface between connective tissue and implants [24, 25].

Our in vitro results show *S. mutans* as an important colonizer that adheres to the abutment surfaces of both titanium and zirconium. A similar result was described in a study performed in vivo with titanium alloy implants coated with titanium nitride (TiN) compared to uncoated titanium implants. After a 24-hour exposure to the oral microbiota, it was found that implants coated with TiN had a smaller amount of surface covered by bacteria from the oral cavity [26]. As for the zirconium abutments, previous studies show that zirconium oxide is a material with a low colonization potential, and even a lower bacterial adherence compared to titanium is described. These results are different from those described in our work where the titanium abutments showed less bacterial adherence compared to the zirconium abutments, but at the same time, greater bacterial viability is described [27, 28].

Other studies performed in vivo using real-time polymerase chain reaction or universal 16S PCR with RFLP obtained very similar results to ours, where it is noted that the surfaces of zirconium oxide and those of titanium alloys are similar in their tendency to adhesion and colonization of *A. actinomycetemcomitans* and *P gingivalis*, both periodontal bacteria that adhere to hard surfaces and soft tissues. However, they only assessed bacterial adherence and not viability [29, 30]. In this context, it is important to emphasize that there is an influence on the bacterial adhesion and on the viability related to the mechanochemical properties of all the components of the implant on different types of surface [31].

On the contrary, there are few studies that describe or determine the bacterial viability on the surface of dental implants; in our work, the bacterial viability was different as far as the pathogen involved in a predominant way. *P. gingivalis* predominated in the titanium pillars and *S. sanguinis* predominated in the pillars of zirconium. In this regard, a study published in 2018 made use of fluorophores, and the processing of images by means of multiphoton microscopy for the analysis of bacterial viability in a heterogeneous population of microorganisms after 48 hours of growth determined that the bacterial viability was similar between the materials of test based on zirconium and titanium [32].

Finally, in the case of the fixation screw, very few studies describe the in vitro adhesion and bacterial viability in the titanium and zirconia fixation screws. According to Dibart et al. and do Nascimento et al., the leakage of bacteria between the abutment and the fixation screw occurs when it is necessary to adjust the screw. However, they recommend further studies to confirm whether the constant adjustment of the fixation screw can increase the incidence of peri-implant disease; in this context, it is important to minimize the presence of bacteria in relation to the abutment-implant connection [33, 34]. In this regard, one study compared the growth of bacterial colonies between anatase-coated titanium healing screws and uncoated titanium healing screws without establishing statistically significant differences in bacterial adhesion and biofilm formation [20, 34]. Perhaps this result is due to the region of the screw evaluated. Scarano et al. evaluated the surface of supra-alveolar screws without finding differences in bacterial colonization between the control groups and the test sample with atanasa, and another is the result when they evaluated the surfaces of the intra-alveolar screws, where the healing screws covered by atanasa present a low colonization potential [35].

In conclusion, our results indicate that there is in vitro bacterial adherence and viability in both titanium and zirconium abutments and fixation screws for both. *Streptococcus mutans* is the microorganism that shows the greatest adherence to the surfaces of both titanium and zirconium and the fixing screws of the latter. On the contrary, bacterial viability is greater on the titanium abutments with *P. gingivalis* than on the zirconium abutments with *S. sanguinis*. With respect to the fixation screws, in both cases, the viability of *S. mutans* was greater with respect to the other bacteria. In general, the titanium abutments showed less adherence but greater bacterial viability.

## 5. Limitations

One of the main limitations of the study was that the physical parameters of the zirconium and titanium surfaces were not evaluated, neither for pillars nor for screws. Furthermore, our results cannot be extrapolated to what could happen in living tissues since it was done in vitro. The sample size was also a limitation since with a larger sample number, we could perform statistical tests. However, we are focused on carrying out additional experiments, taking into consideration various parameters and carrying out experiments in vivo in the future.

## Data Availability

The data used to support the findings of this study are available from the corresponding author upon request.
